# Effectiveness and safety of SR-ENS-600endoscopic surgical system in benign and malignant gynecological diseases: a prospective, multicenter, clinical trial with 63 cases

**DOI:** 10.1007/s11701-024-01871-4

**Published:** 2024-05-10

**Authors:** Ren Chang, Duan Ping, Shen Yang, Wang Yongjun, Zhang Wei, Zheng Ying, Li Xingming, Zhang Kexin, Sun Dawei

**Affiliations:** 1 Department of Obstetrics and Gynecology, State Key Laboratory of Complex Severe and Rare Diseases, National Clinical Research Center for Obstetric & Gynecologic Diseases, Peking Union Medical College Hospital, Chinese Academy of Medical Sciences and Peking Union Medical College, Beijing, 100730 China; 2 Department of Obstetrics and Gynecology, Oncology Discipline Group, The Second Affiliated Hospital of Wenzhou Medical University, Wenzhou, 325027 China; 3 Department of Obstetrics and Gynecology, School of Medicine, Zhongda Hospital, Southeast University, Nanjing, 210009 China; 4 Department of Obstetrics and Gynecology, Beijing Jishuitan Hospital, Capital Medical University, Beijing, 100035 China; 5 Department of Obstetrics and Gynecology, Zhongnan Hospital of Wuhan University, Wuhan, 430071 China; 6 Department of Gynecologic Oncology, Key Laboratory of Birth Defects and Related Diseases of Women and Children, Sichuan University West China Second University Hospital, Chengdu, 610041 China; 7 School of Public Health, Capital Medical University, Beijing, 100069 China

**Keywords:** Single-incision surgery, Single-port laparoscopic surgery, Single-port robotic laparoscopic surgery, Minimally invasive surgery

## Abstract

Single-port laparoscopy has gained more attention, but inherent technical challenges hinder its wider use. To overcome the disadvantage of traditional single-port surgery, robotic laparoendoscopic single-site surgery system was designed and clinically utilized. This multi-center single-arm trial was aimed to present the clinical outcomes of the SHURUI robotic endoscopic single-site surgery system. 63 women with ovary cysts, myoma, cervical epithelial neoplasm, or endometrial carcinoma were recruited at 6 academic medical centers in different districts of China. The trial was registered on September 5, 2023, with the register number: ChiCTR2300075431, retrospectively registered. Patients underwent robotic LESS surgery with the SHURUI endoscopic surgical system from January 17 to May 26, 2023. Demographic information, perioperative parameters, complications, scar healing, and operator satisfaction scores were recorded. Patients were followed up for 30 ± 4 days. Average operative time and estimated blood loss were 157.03 ± 75.24 min and 63.86 ± 98.33 ml, respectively, for all surgeries. Average anal exhaust time and hospitalization stay were 30.99 ± 14.25 h and 3.63 ± 1.59 days, respectively. Patients’ postoperative rehabilitation assessment showed satisfactory results on the day of discharge and 30 ± 4 days after surgery. The surgery achieved good cosmetic benefits and was surgeon friendly. There were no conversions to alternative surgical modalities, complications, or readmissions. The SHURUI endoscopic surgical system showed both the technical feasibility and safety of this surgical modality for gynecologic patients. Further randomized studies comparing this modality with traditional LESS surgery are suggested.

## Introduction

Within the last 10 years, significant advances in minimal-invasive surgery have been reported. Due to the advantages of improved cosmesis, fewer complications, less pain, shorter hospital stays, and lower health care costs, single-port laparoscopy has gained increasing attention. Sixty-four percent of gynecological patients preferred the appearance of a single-port laparoscopic scar when surveyed [1]. However, the operation is challenging for surgeons owing to technical and ergonomic difficulties, including loss of instrumental triangulation, reduced operative working place, blocked visualization, instrumental crowding, and clashing [2].

A robotic platform was thus developed to help restore intra-abdominal triangulation while maintaining the maximum degree of freedom for precise maneuvers. Significant advances have been achieved in the field of robotic laparoendoscopic single-site (R-LESS) surgery since the first reported clinical series in 2009. The specially designed da Vinci single-port surgical robot system (SP) produced by the Intuitive Surgical was approved for clinical application by the United States Food & Drug Administration (FDA) in 2018. Its use in gynecological surgery was relatively recent compared to urology and general surgery; moreover, it has not yet been allowed to be exported to China. The endoscopic surgery system was developed by Beijing Surgerii Robotics Co., Ltd. and is a single-port surgical robot system independently developed in China.

To explore the effectiveness and the safety of the SHURUI endoscopic surgical system (SR-ENS-600) in gynecological diseases, a prospective, multicenter, single-arm clinical trial was carried out, and the results are reported below.

## Materials and methods

### Ethics statement and registration

This prospective, multicenter, single-arm clinical trial was conducted between January 17 and May 26, 2023. The institutional review board approved the study (K2022566) on September 8, 2022. The trial was registered at https://www.chictr.org.cn/ (registration number: ChiCTR2300075431).

### Patient population

Eligible patients with ovary cysts, myoma, cervical epithelial neoplasms, and endometrial carcinoma were recruited according to the following inclusion and exclusion criteria.

Inclusion Criteria

1. Females, aged between 18 and 75 years;
2. Patients with indications for elective gynecological surgery;
3. Patients who can tolerate surgery and anesthesia;
4. Patients without a history of complicated gynecological diseases and serious diseases;
5. Patients with no history of mental illness;
6. Patients with body mass index (BMI) ≤ 32 kg/m^2^;
7. Patients who agreed to the study, had good compliance, and voluntarily signed an informed consent form.

Exclusion criteria.

1. Patients with a history of more than two pelvic or abdominal surgeries or severe pelvic adhesions;
2. Patients with ruptured ectopic pregnancy, massive bleeding and unstable vital signs;
3. Patients with acute genital tract or systemic infection;
4. Patients with coagulation dysfunction or treated with long-term anticoagulant drugs;
5. Patients with severe heart and lung function diseases, liver and kidney insufficiency, or unable to tolerate anesthesia;
6. Patients with abdominal wall or diaphragmatic hernia, abnormal umbilical development, or history of umbilical surgery;
7. Patients who refuse to undergo laparoscopic surgery;
8. Patients who had participated in clinical trials of other drugs or devices within 3 months before surgery.

### Study design

The sample size was calculated before the study commenced. According to clinical experience, P0 for this experimental device was set as 90%, and the expected surgical success rate PT was 99%. The power (1-β) was 80% and *α* was 0.025 (unilateral); 56 women would be needed to observe a significant difference in primary outcome according to calculation. The sample size was increased by 10% to account for potential dropout; thus, the final sample size was 63 women.

**Fig. 1:**
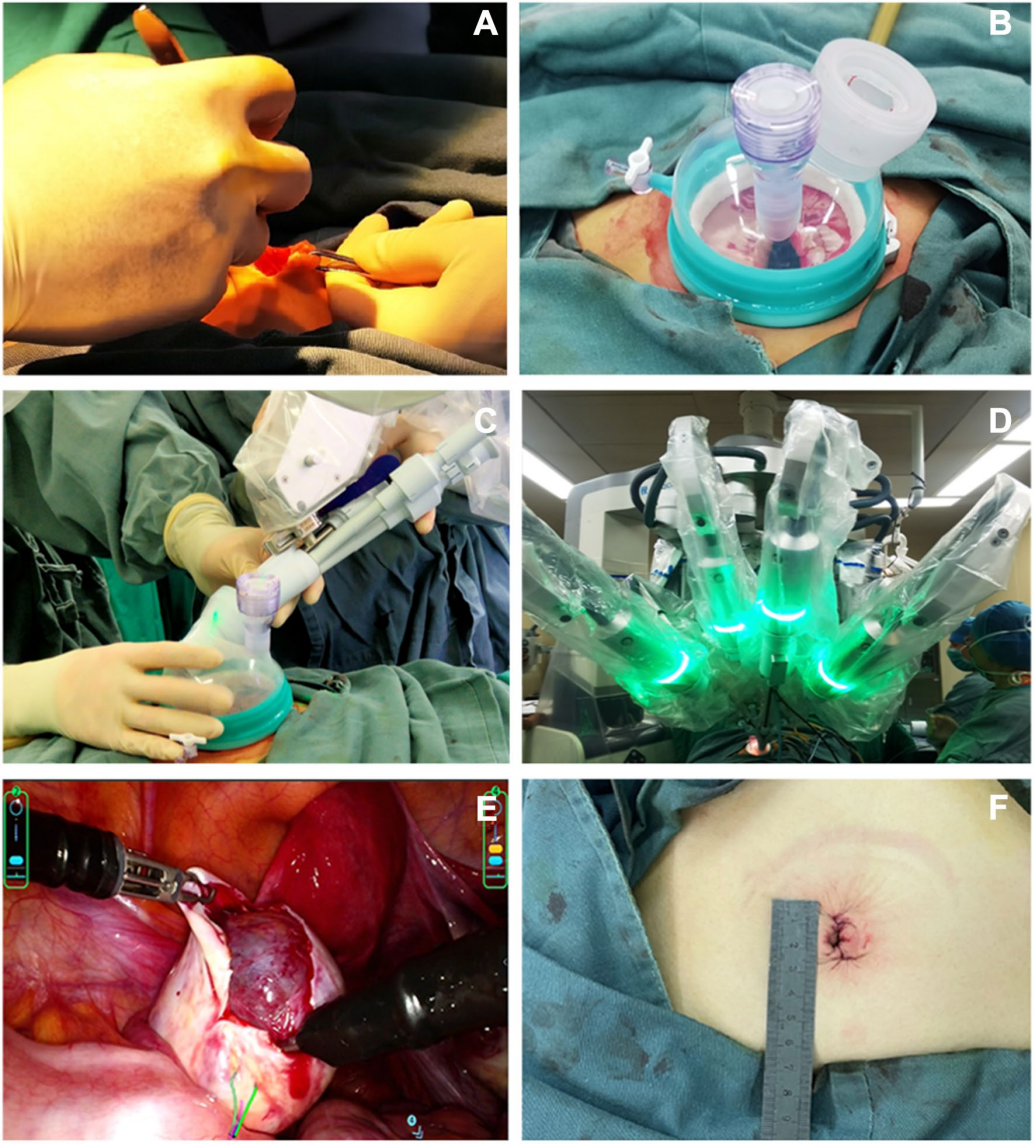
Surgical procedure of the operation **A** Making a transumbilical incision through the Hasson approach, **B** placement of the surgical port, **C** insertion of the four-channel trocar, **D** docking, **E** dissection of the ovary cyst, **F** closure of the umbilical incision.

Written informed consent was obtained from all participants before their enrollment, and all practices were performed according to related regulations. Medical history, vital signs, general and gynecological physical examination, laboratory tests, and imaging examination were performed. All surgical procedures were performed by one gynecologist who was both experienced in traditional single-port surgery and robotic endoscopic multiport surgery system in each center. Simply put, the SHURUI endoscopic surgical system consists of a surgeon console and a surgical trolley, a 3D endoscope and several surgical instruments. A trans-umbilical incision of approximately 25 mm (as measured by a sterile ruler) was made through an open Hasson approach before a four-channel trocar was inserted (see Fig. 1A–C). The patient was placed in a steep Trendelenburg position, and the surgical trolley was subsequently parked beside the patient-side bed and the robot was docked on the patient’s left side. The surgical instruments and the 3D endoscope were then loaded on the surgical trolley and steered through the access channels in the trocar to enter the patient’s abdomen (see Fig. 1D). The surgeon then accomplished the surgery by maneuvering the haptic devices on the surgeon console to tele-operate the surgical instruments or the 3D endoscope under the guidance of stereo visual feedback of the surgical site (see Fig. 1E). During the teleoperation, the surgical instruments were controlled in an intuitive manner to reduce the mental work of the manipulation. The incision was closed with purse-string suture of both peritoneum and rectus sheath, and then continuous suture of subcutaneous tissue and intradermic suture (see Fig. 1F).

Demographic information, perioperative parameters, and complications—including adjacent organ injury, fever, surgical field infection, pelvic hematoma, thromboembolism, and postoperative intestinal obstruction—as well as scar healing and operator satisfaction scores were recorded.

### Outcomes

The primary outcome was the surgical success rate. Surgical success is defined as no conversion to open surgery or other conventional endoscopic surgeries for any reason. Surgical success rate = number of subjects operated on as planned/total number of included subjects × 100%.

Secondary evaluation parameters included operation time, bleeding volume, hospitalization stay, anal exhaust time, incision healing grade (evaluated at discharge day and 30 ± 4 days after surgery) (see Table 1), incision scar satisfaction score evaluated with The Scar Cosmesis Assessment and Rating (SCAR) scale [3] (30 ± 4 days after surgery) (see Table 2), subjective postoperative rehabilitation score (discharge day, 30 ± 4 days after surgery), surgeon satisfaction score and safety evaluation parameters: laboratory examination, vital signs, reoperation rate, perioperative complications, and AE/SAE status. Scar-related parameters were measured by trained nurses, and the subjective postoperative rehabilitation score, surgeon satisfaction score, and safety evaluation were self-reported using a standard questionnaire.

**Table 1:** Incision healing grade

Incision healing grade	Grade parameter
A	Excellent healing, no redness and infection at the incision, linear healing
B	Healing is poor, the incision has an inflammatory response but is not suppurated, and the incision is partially linear
C	The incision skin is large and suppurated, requiring incision drainage

**Table 2:** The Scar Cosmesis Assessment and Rating (SCAR) scale

Parameter	Descriptor	Score
Clinician questions		0
Scar spread	None/near invisible	0
	Pencil-thin line	1
	Mild spread, noticeable on close inspection	2
	Moderate spread, obvious scarring	3
	Severe spread	4
Erythema	None	0
	Light pink, some telangiectasias may be present	1
	Red, many telangiectasias may be present	2
	Deep red or purple	3
Dyspigmentation	Absent	0
	Present	1
Suture marks	Absent	0
	Present	1
Hypertrophy/atrophy	None	0
	Mild: palpable, barely visible hypertrophy or atrophy	1
	Moderate: clearly visible hypertrophy or atrophy	2
	Severe: marked hypertrophy or atrophy or keloid formation	3
Overall impression	Desirable scar	0
	Undesirable scar	1
Patient questions		
Itch	No	0
	Yes	1
Pain	No	0
	Yes	1

Subjective postoperative rehabilitation scores were evaluated with the following questions scaled between 0 and 10 according to severity: 1. breathing well, 2. good appetite, 3. feeling relaxed, 4. sleeping well, 5. able to use the toilet and take care of personal hygiene by herself, 6. able to communicate with family or friends, 7. able to communicate with doctors and nurses and get their support and encouragement, 8. able to engage in work or family activities, 9. feeling comfortable and self-controlling, 10. feeling healthy overall, 11. moderate pain, 12. intense pain, 13. nausea and vomiting, 14. feeling nervous and anxious, and 15. feeling sad and depressed within the past 24 h. The postoperative rehabilitation score consists of two parts: the higher the comfort score (questions 1–10), the better, with a maximum score of 100; the lower the scores for pain and anxiety (questions 11–15), the better, 0 means no above symptoms present, with a total score of 50.

The Surgeon Satisfaction Questionnaire included two parts: system performance (questions 1–12) and operator comfort-related scores (questions 13–20), with 1–5 points from most uncomfortable/difficult to most comfortable/easy for each question and a full score of 100 points.

### Statistical analysis

Statistical analysis was carried out using SPSS Statistics for Windows (version 25.0; SPSS Inc., Chicago, IL, USA), and the sample size calculation adopted PASS 13 (NCSS, LLC). The curative effect evaluation included the analysis of the PPS and FAS sets, and the conclusion was mainly based on the FAS set. The safety evaluation adopted SS set analysis. The descriptive statistical analysis of quantitative indicators was reported as the number of cases, mean, standard deviation, median, lower quartile (*Q1*), and upper quartile (*Q3*), and the statistical description of categorical variables included frequency and percentage. A one-sample *t* test was used to compare the difference between the results from the present study and the literature using the da Vinci SP system performing same surgery as in our study. The results were deemed significantly different from each other when the two-sided P value was less than 0.05.

### Quality control

Before the study began, relevant training was provided to all researchers participating in the study to ensure that they fully understood the process. All investigators authorized to perform surgeries in participating research centers had relevant surgical experience to reduce the bias caused by the researcher’s skills on the trial results. All independent evaluators received unified training to reduce the impact of differences in evaluators' evaluations on the trial results. When the clinical study was completed, data storage and organization were ensured. The data administrator checked and confirmed the data through the data clarification form to avoid recording errors.

## Results

### Participant disposition and demographics

**Fig. 2:**
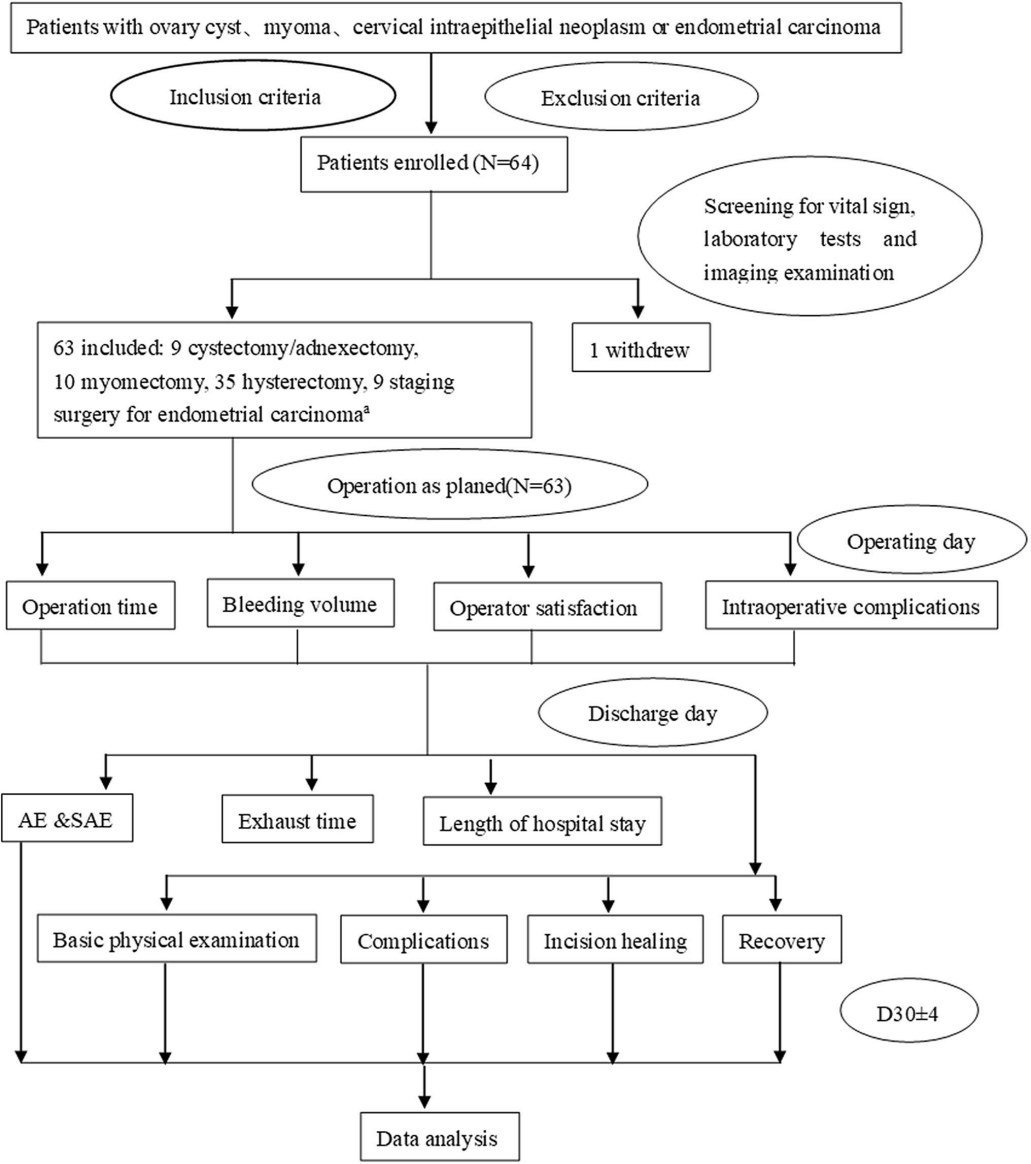
Participant flow chart ^a^Eight patients underwent total hysterectomy + bilateral adnexectomy + pelvic sentinel lymphadenectomy, and 1 patient received total hysterectomy + bilateral adnexectomy + pelvic lymphadenectomy.

A total of 64 cases were enrolled in 6 hospitals in different districts of China, with 11 cases enrolled in center 1, 16 cases in center 2, 10 cases in center 3, 7 cases in center 4, 10 cases in center 5, and 12 cases in center 6. One subject voluntarily withdrew from the trial due to psychological stress and preoperative anxiety and thus was excluded from all datasets (FAS, PPS, and SS), with 63 cases included in the final datasets (Fig. 2).

**Fig. 3:**
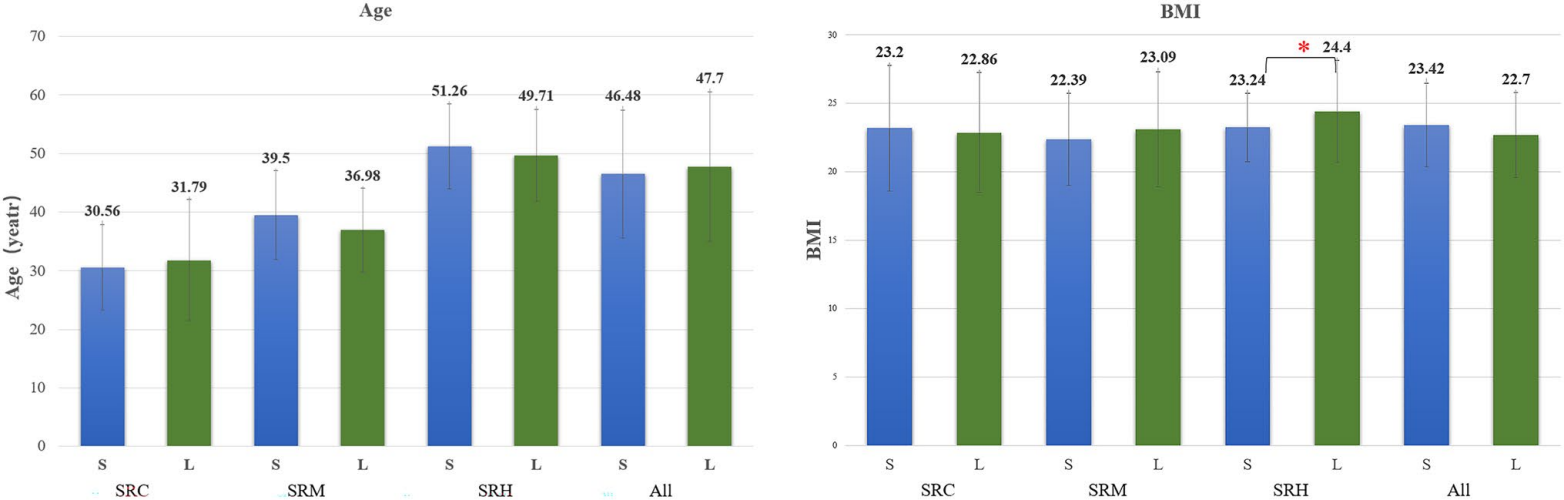
Demographic information of the patients compared with the literature^4,5^ SRC stands for single-port robotic cystectomy/adnexectomy, SRM stands for single-port robotic myomectomy, SRH stands for single-port robotic hysterectomy, All stands for all surgery. S represents the results of the present study, and L represents the results from the literature.

Sixty (95.2%) patients were of Han nationality, and 37 (58.7%) patients had a previous history of surgery. Nineteen (30.2%) patients were postmenopausal for 7.00(4.00, 13.00) years. Fourteen (31.8%) patients had a history of dysmenorrhea. Gestation and parturition for all patients were 3.00 (1.00, 4.00) and 1.00 (0.00, 2.00) respectively. The patient withdrawn was 33 years old and her BMI was 20.3 kg/m^2^, she suffered from dysmenorrhea and had one abortion before. Nine (14.3%) patients received cystectomy/adnexectomy, 10 (15.9%) underwent myomectomy, 35 (55.6%) received hysterectomy, and 9 (14.3%) patients underwent staging surgery for endometrial carcinoma. Other demographic information of the patients compared with the literature [4, 5] is shown in Table 3 and Fig. 3.

**Table 3:** Demographic information of the patients compared to the literature [4, 5]

Variables^a^	SRC			SRM			SRH			SRES	All		
	S (*n* = 9)	L^4^(*n* = 108)	*p* value	S (*n* = 10)	L^4^ (*n* = 56)	*p* value	S (*n* = 35)	L^4^ (*n* = 35)	*p* value	S (*n* = 9)	S (*n* = 63)	L^5^ (*n* = 31)	*p* value
Age (year)	30.56 ± 7.25	31.79 ± 10.31	0.623	39.50 ± 7.52	36.98 ± 7.14	0.317	51.26 ± 7.27	49.71 ± 7.86	0.217	51.56 ± 10.32	46.48 ± 10.91	47.7 ± 12.8	0.377
BMI (kg/m^2^)	23.20 ± 4.60	22.86 ± 4.39	0.830	22.39 ± 3.37	23.09 ± 4.19	0.527	23.24 ± 2.50	24.40 ± 3.74^4^	0.010^b^	25.49 ± 2.37	23.42 ± 3.06	22.7 ± 3.1	0.065

^a^Data are presented as the mean ± standard deviation, median (*Q1*, *Q3*), or number (percentage). S stands for results of present study, L stands for results from the literature, W stands for results of withdrawn patient. ^b^stands for results showing differences between the present and literature

### Surgical and safety profiles of patients

The surgical success rate was 100.0% (95% CI: 94.3–100.0%).

**Fig. 4:**
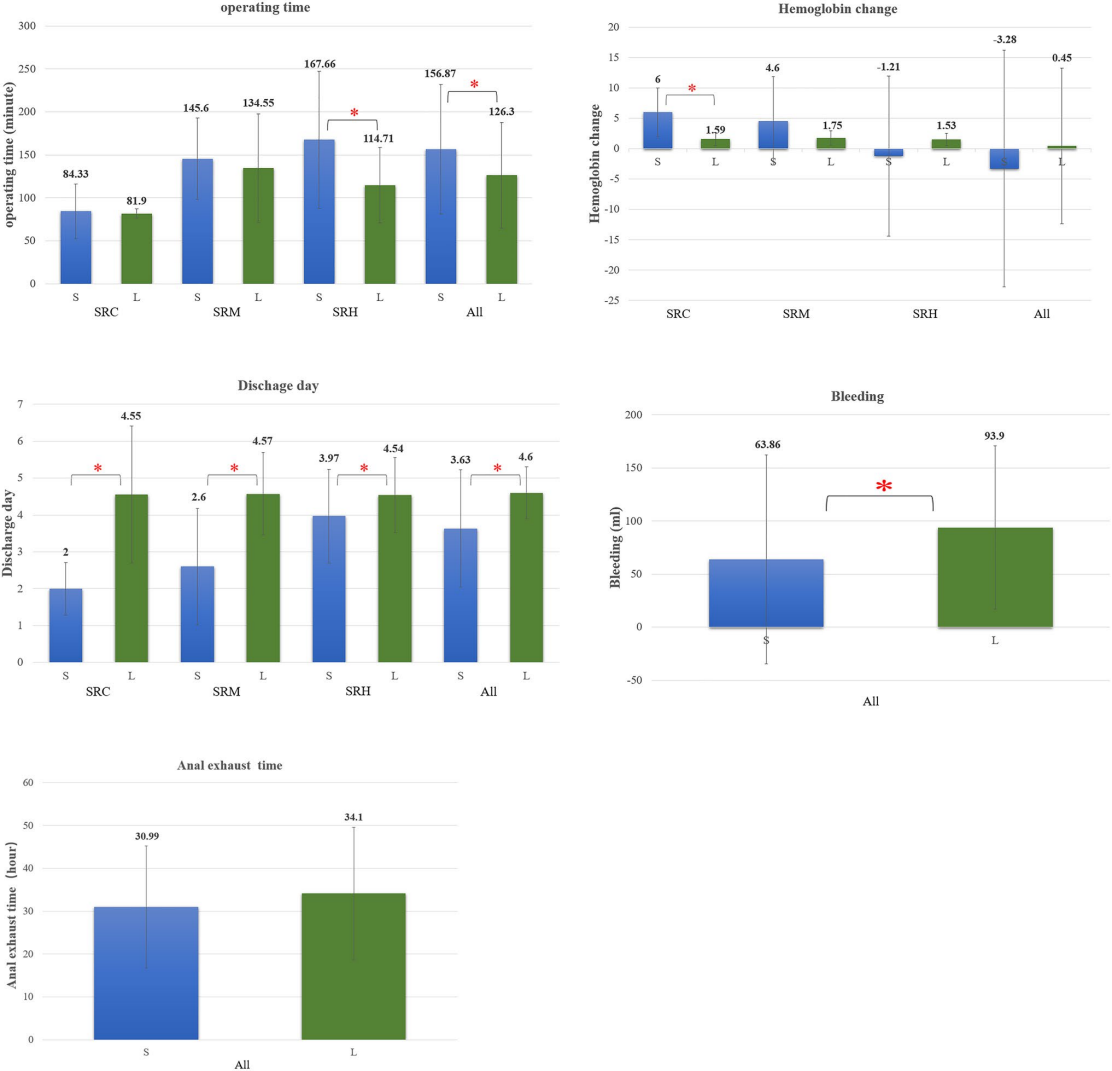
Perioperative indicators of the patients compared to the literature^4,5^ SRC stands for single-port robotic cystectomy/adnexectomy, SRM stands for single-port robotic myomectomy, SRH stands for single-port robotic hysterectomy, All stands for all surgery. S represents the results of the present study, and L represents the results from the literature.

Perioperative indicators of the patients compared to the literature [4, 5] are shown in Table 4 and Fig. 4.

**Table 4:** Perioperative indicators of the patients compared to the literature [4, 5]

Variables	SRC			SRM			SRH			SRES	All		
	S(*n* = 9)	L^4^ (*n* = 108)	*p* value	S (*n* = 10)	L^4^ (*n* = 56)	p value	S (*n* = 35)	L^4^ (*n* = 35)	*p* value	S (*n* = 9)	S (*n* = 63)	L^5^ (*n* = 31)	*p* value
Operation time (min)	84.33 ± 31.95	81.90 ± 45.07	0.825	145.60 ± 47.38	134.55 ± 63.39	0.480	167.66 ± 79.65	114.71 ± 44.20	0.000^a^	200.00 ± 69.33	156.87 ± 75.34	126.3 ± 61.6	0.002^a^
Bleeding volume (ml)	12.00 ± 9.58			51.50 ± 49.44			80.86 ± 124.77			63.33 ± 38.08	63.86 ± 98.33	93.9 ± 77.0	0.018^a^
Hemoglobin change (g/l)	6.00 ± 4.03	1.59 ± 1.08^4^	0.011^a^	4.60 ± 7.28	1.75 ± 1.20	0.247	−1.21 ± 13.18	1.53 ± 1.04	0.227	-3.28 ± 19.52	0.45 ± 12.85	2.4 ± 0.8	0.232
Anal exhaust time (hr)	21.86 ± 9.96			23.76 ± 6.35			35.01 ± 14.81			32.49 ± 16.24	30.99 ± 14.25	34.1 ± 15.5	0.088
Hospitalization stay(day)	2.00 ± 0.71	4.55 ± 1.86	0.000^a^	2.60 ± 1.58	4.57 ± 1.12	0.003^a^	3.97 ± 1.27	4.54 ± 1.01	0.012^a^	5.11 ± 1.45	3.63 ± 1.59	4.6 ± 0.7	0.000^a^

*SRC* stands for single-port robotic cystectomy/adnexectomy, *SRM* stands for single-port robotic myomectomy, *SRH* stands for single-port robotic hysterectomy, *SRES* stands for single-port robotic endometrial carcinoma staging. *S* represents the results of the present study, and *L* represents the results from the literature. ^a^ indicates results showing differences between the present study and the literature

All patients experienced grade A incision healing on the discharge day and D30 ± 4 after surgery. The incision scar satisfaction score evaluated with the Scar Cosmesis Assessment and Rating (SCAR) scale was no more than 1 point for all patients, with over 55.6% of patients scoring 0 for all items except for scar spread after myomectomy.

On the day of discharge and D30 ± 4 after surgery, the average postoperative rehabilitation assessment scores were 88.71 ± 10.64 points and 94.87 ± 8.16 points, respectively, and the pain and anxiety scores were 3.38 ± 3.18 points and 0.87 ± 2.88 points, respectively.

Regarding the surgeon satisfaction score, the average score related to system performance was 51.35 ± 6.73 points, the average score related to operator comfort was 35.29 ± 4.03 points, and the average total score was 86.63 ± 10.59 points. Approximately 85% of surgeons gave 3 ~ 5 points for each item.

No patients suffered from intraoperative or postoperative complications, and no patients experienced reoperation on D30 ± 4. There were 79 mild adverse events, 9 moderate adverse events (including 3 cases of nausea and vomiting, 2 cases of anemia, 2 cases of acute respiratory infection, 1 case of vaginal bleeding, and 1 case of insomnia) and 1 severe adverse event (vaginal stump bleeding after discharge needing resuturing) reported, but none were trial-related. Compared with rigid links, the continuum had softer gripping power in pulling out fibroids.

## Discussion

Minimally invasive surgery has revolutionized the treatment of gynecologic diseases over the last 30 years. The advantages of laparoscopic surgery over abdominal surgery have been well established [6, 7]. However, there are several limitations to traditional laparoscopy, including counterintuitive hand movements, accentuated tremors and ergonomically challenging positions, which may result in fatigue and even injuries to the surgeon with lengthy practice. With the advent of the da Vinci robotic system (DRS), many surgeries are now being performed with robotic procedures. The rate of robotic surgeries performed in 2012 was significantly increased by 50% compared to 27.9% performed in 2007 [8].

**Fig. 5:**
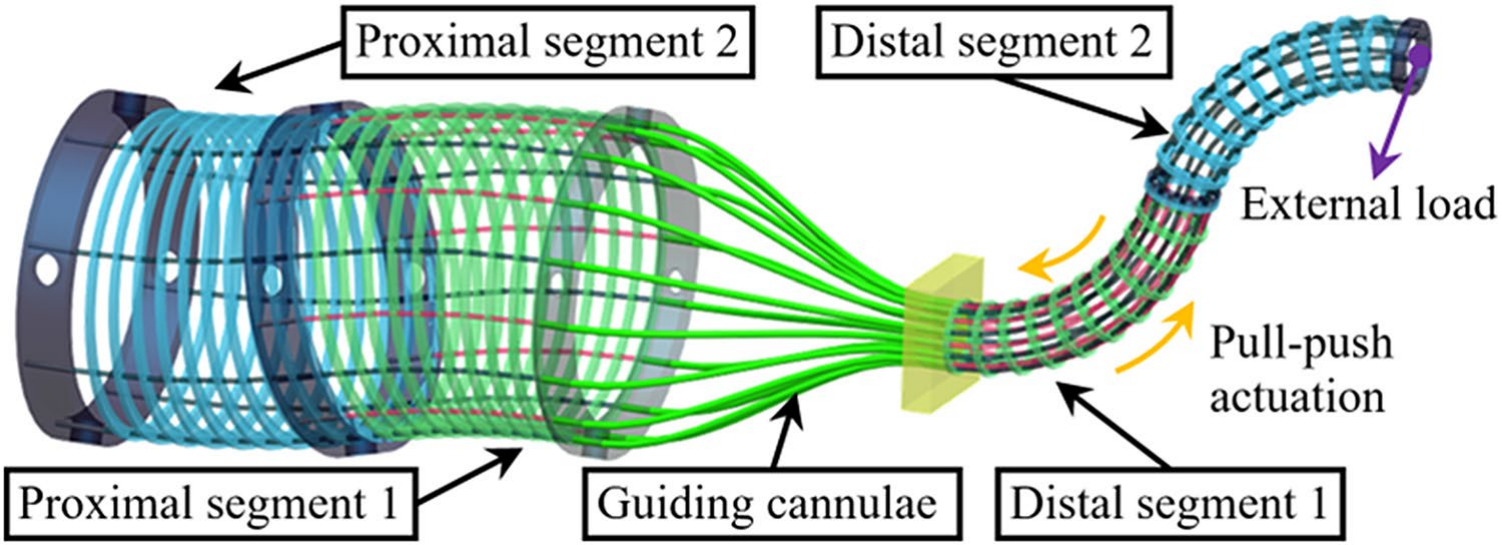
Dual continuum mechanism

In the meantime, laparoendoscopic single-site surgery has shown great attraction owing to its cosmetic benefits; however, it has surgical limitations in angulation and device manipulation [9]. Robotic single-port surgery, on the other hand, combines the advantages of robotics with the esthetic result of a single incision. In the literature, compared with LESS hysterectomy, R-LESS had a longer operating time but had less blood loss, a decreased length of hospital stays, and a favorable learning curve [10, 11]. In the SHURUI endoscopic surgical system, both the robotic instrument and the 3D endoscopy are composed of a so-called dual continuum mechanism. Unlike conventional instruments that are composed of joints and rigid links, the continuum mechanism achieves motion via continuous deformation of the elastic structure and is designed for enhanced payload capability. As shown in Fig. 5, the dual continuum mechanism consists of proximal segments, distal segments and guiding cannula. The bending of the proximal segment is coupled to that of the distal segment via the translations (pulling and pushing) of all the structural backbones made of super-elastic Ni–Ti alloy (Fig. 5). The failure of several backbones does not affect the functionality of the mechanism. And the backbones could collaboratively balance the external load applied on the segment. Therefore, the dual continuum mechanism in the SHURUI endoscopic surgical system can help realize a payload-enhanced multi-DoF surgical instrument with a tight bending wrist for intra-abdomen dexterity. The advantages of such structure include flexible arms, compact size, high accessibility with sufficient triangulation and motion degree of freedom [12].

In the present trial, the demographic information of patients was similar to that of the other two studies using the da Vinci SP [4, 5], except that the BMI of patients who received hysterectomy in our study was lower than that of Kim’s study (23.24 ± 2.50 vs. 24.40 ± 3.74, *p* = 0.010) [4].

In regard to perioperative parameters, the operating time showed no difference for cystectomy/adnexectomy and myomectomy but was longer for hysterectomy in our study (167.66 ± 79.65 vs. 114.71 ± 44.20 min, *p* < 0.001) [4]. The possible reason for this was because one center included patients with multiple-myoma, especially one nullipara patient with both adenomyosis and myoma whose uterus was irregularly enlarged, parametrium was severely shortened and lacked mobility, with severe uterosacral ligament adhesion. The whole surgery took 472.00 min, and the bleeding volume was 700 ml. This might account for the prolonged mean operating time for hysterectomy. This also led to a longer mean operating time in all surgeries compared with that in Shin’s study (156.87 ± 75.34 vs. 126.3 ± 61.6 min, *p* = 0.002) [5]. After cystectomy/adnexectomy, hemoglobin was reduced more than that reported by Kim [4]. Almost all surgeons who started with cystectomy/adnexectomy for practice might account for this result, and we noticed that as the trial went on, there was less bleeding with cystectomy/adnexectomy. We also found that there was less bleeding in the whole group than in the Shin group (63.86 ± 98.33 vs. 93.9 ± 77.0 ml, *p* = 0.018) [5], but the hemoglobin change showed no difference. The anal exhaust time for all surgeries including gynecologic tumors was 30.99 ± 14.25 h in the present trial compared to 34.1 ± 15.5 h reported by Shin, but there was no significant difference (*p* = 0.088) [5]. The most notable difference between our results and the literature was a much shorter hospital stay for all surgery types (*p* < 0.015), which was an important parameter for postoperative rehabilitation [4, 5].

There was no conversion to multiport laparoscopy or laparotomy or perioperative complications in our and other studies, except that Kwak reported 1 case (1.3%) of superficial bowel laceration due to severe adhesion from prior surgery during endometrial cancer staging and 1 case (1.3%) of wound complication after discharge [4, 5, 13–15].

The SHURUI endoscopic surgical system was shown to be efficacious and safe in cystectomy/adnexectomy, myomectomy, hysterectomy, or endometrial carcinoma staging under the conditions of this study.

Since this study was conducted under careful quality control, the results suggest that ovary cysts, myoma, cervical epithelial neoplasms, and endometrial carcinoma may be appropriate indication for the SHURUI endoscopic surgical system. Randomized controlled trials are needed to determine whether the SHURUI endoscopic surgical system is effective for a wider range of gynecological indications.

The present trial has several notable features. To our knowledge, this is the first report of gynecological results using the SHURUI endoscopic surgical system and the first study to include both benign and malignant gynecologic disorders. To explore its benefits as a minimally invasive surgery, we evaluated the results of incision healing and scar cosmesis assessment, which revealed that all patients experienced grade A healing, and over 65% of patients showed no scar on D30 ± 4. With 100% of patients scoring no more than 1 point, when concealed in the umbilicus, the incision successfully satisfied patients’ demand for cosmesis. Furthermore, an ideal single-port robotic surgery system should allow good visualization, prevent hand tremors, provide three-dimensional (3D) high-definition images, and allow the surgeon to perform lengthy surgery in a more comfortable position. Thus, our study evaluated the surgeon’s satisfaction score and found that the average scores related to system performance and operator comfort were 51.35 ± 6.73 out of 60 points and 35.29 ± 4.03 points out of 40 points, respectively, with a total score of 86.63 ± 10.59 out of 100 points, which showed good experience with the surgeons. The prospective, multicenter nature of this study avoided the possible limitations of a single research institution and restricted population, making its results more generalizable. Careful sample size calculation and strict quality control ensured the reliability of the results.

The greatest limitation of this study was its single-arm design, which hinders direct comparison with traditional and multiport robotic laparoscopic surgery. Further randomized controlled trials are needed to confirm the present results. Another limitation was that one-sample t test was not appropriate to calculate variables such as percentage of dysmenorrhea or numbers of gestation and parturition, and thus we could not identify if this demographic information was comparable to literature.

## Conclusions

The clinical outcomes of single-port laparoscopic surgery using the SHURUI endoscopic surgical system for gynecological disorders, including cystectomy/adnexectomy, myomectomy, hysterectomy, and endometrial carcinoma staging, were comparable to those performed with the da Vinci robotic single-port system. There was no need for conversion to multiport or open surgery, nor was there any operative complications. It achieved good cosmetic benefits and was surgeon-friendly.

Therefore, this study suggests that the SHURUI endoscopic surgical system can be proposed as a relatively safe and feasible surgical method for patients who desire to undergo single-port surgery.

## Data Availability

The data is available upon request.
